# Resection of Hilar Cholangiocarcinoma

**DOI:** 10.1155/1998/24893

**Published:** 1998

**Authors:** Steven M. Strasberg

**Affiliations:** Washington School of Medicine 1 Barnes Hospital Plaza Box 8109 St Louis M0 63110 USA

## Abstract

*Objective*: Morbidity and mortality involved in the resection of hilar cholangiocarcinoma were reviewed retrospectively. The clinicopathologic and laboratory parameters that might influence the patient's survival also were re-evaluated.

*Summary Background Data*: Although much progress has been made in the diagnosis and management of hilar cholangiocarcinoma, long-term outlook for most patients remains poor. Surgical resection is usually prohibited because of its local invasiveness, and most patients can only be managed by palliative drainage. Recently, many surgeons have adopted a more aggressive resection with varying degrees of success. Several prognostic factors in bile duct carcinoma have been proposed; however, no reports have specifically focused on resected hilar cholangiocarcinoma and its prognostic survival factors using multivariate analysis.

*Methods*: The clinical records and pathologic slides of 49 cases with resected hilar cholangiocarcinoma were reviewed retrospectively. Twenty clinical and laboratory parameters were evaluated for their correlation with postoperative morbidity and mortality, whereas 31 variables were evaluated for their significance with postoperative survival. Variables showing statistical significance in the first univariate analysis were included in the following multivariate analysis using stepwise logistic regression test for factors affecting morbidity and mortality and Cox stepwise proportional hazard model for factors influencing survival.

*Results*: There were 5 in-hospital deaths, and the cumulative 5-year survival rate in 44 patients who survived was 14.9%, with a median survival of 14.0 months. Multivariate analysis disclosed that coexistent hepatolithiasis and lower serum asparate aminotransferase levels (90 U/L) had a significant low incidence of postoperative morbidity, whereas a serum albumin of less than 3 g/dL was the only significant factor affecting mortality. Regarding survival, univariate analysis identified eight significant factors: 1) total bilirubin 10 mg/dL, 2) curative resection, 3) histologic type, 4) perineural invasion, 5) liver invasion, 6) depth of cancer invasion, 7) positive proximal resected margin, and 8) positive surgical margin. However, multivariate analysis disclosed total bilirubin ≥ 10 mg/dL, curative resection, and histologic type as the three most significant independent variables.

*Conclusions*: Surgical resection provides the best survival for bilar cholangiocarcinoma. An adequate nutritional support to increase serum albumin over 3g/dL is the most important factor to decrease postoperative mortality. Moreover, preoperative biliary drainage to decrease jaundice and a curative resection with adequate surgical margin are recommended  if longer survival is anticipated. Patients with well differentiated adenocarcinoma seem to survive longer compared to those with moderately or poorly differentiated tumors.


The authors of this paper report a 5-year study on 57 patients, using a polytetrafluoroethylene (PTFE), small-diameter graft between the superior mesenteric vein and the inferior cava. These operations were carried out when sclerotherapy had failed, failure being defined as two recurrences of bleeding from the oesophageal varices or one recurrence from gastric varices. The reported results seem good, with only 3 deaths (5%) in the post-operative period, and only 2 recurrences of haemorrhage in the immediate post-operative period and one recurrent later bleed in the second year after operation. The incidence of encephalopathy was reported as 9 per cent, and was managed successfully by protein restriction and/or lactulose treatement. The cumulative patency rate of the shunt was 95 per cent. There was a 75 per cent actuarial survival rate at 6 years.

The patients were extensively investigated before, and after, operation. The operative techniques were standard apart from the use of a PTFE graft. The patients were put on to heparin initially intravenously and later subcutaneously, to maintain a clotting level of twice normal. Significant reduction in portal pressure was achieved with shunt diameters of 10 and 12 millimetres. Prograde hepatic flow was preserved for at least 3 years. Chronic encephalopathy did not feature after shunting, but detailed description of the results of pre- and post-operative tests for encephalopathy were not provided.

The main criticism of the paper is that the study was restricted to patients who had good liver function, mainly Child-Pugh grade A and B. In the experience of most clinicians in this field, the majority of patients belong to the Child-Pugh grade C. How did the authors treat those in Child-Pugh grade C? Moreover, the authors do not describe how they assessed the severity of haemorrhage preceding these operations. Were these severe haemorrhages or mild, but repeated, bleeds? There is good evidence that the incidence of hepatic failure and encephalophy after any type of shunt is remarkably low in patients who have good liver function and who have not bled severely.

There is no doubt that the interposition mesocaval shunt is the easiest of all shunts to perform. The shunt can be easily taken down if, and when, liver transplant has to be undertaken. The authors have an extensive experience in this field, and have contributed significantly to the surgical literature. However, since the early days of end-to-side portacaval shunt, the tale has always been the same. The first reports of a new treatment have been most encouraging. The initial studies have been undertaken in a small number of highly-selected patients by an enthusiastic dedictated group. Only when the new treatment has been undertaken in larger numbers, in a less selected group of patients, and reported by a variety of groups, has pessimism set in.

The use of PTFE graft for mesocaval interposition, therefore, is a useful technique for the surgeon to keep at the back of his mind, for it is a simple technique when the superior mesenteric vein and the inferior vena cava are both patent. The results of this paper apply only to a small proportion of patients with bleeding oesophageal varices, and it has not been proved that the results are better than other forms of shunt. The authors are invited to undertake a randomised control trial comparing this technique with other established procedures.

Professor Sir Robert Shields
Oce of the President
The Royal College of Surgeons
of Edinburgh
Nicolson Street
Edinburgh EH8 9DW
United Kingdom

### Resection of Hilar Cholangiocarcinoma

### ABSTRACT

*Su, C.-H., Tsay, S.-H., Wu, C.-C., Shyr, Y.-M., King, K.-L., Lee, C.-H., Lui, W.-Y., Liu, T.-J. and P'eng, F.-K. (1996) Factors influencing postoperative morbidity, mortality and survival after resection for hilar cholangiocarcinoma. Annals of Surgery, 223, 384–394.*

***Objective:*** **Morbidity and mortality involved in the resection of hilar cholangiocarcinoma were reviewed retrospectively. The clinicopathologic and laboratory parameters that might influence the patient's survival also were re-evaluated.**
***Summary Background Data:*** **Although much progress has been made in the diagnosis and management of hilar cholangiocarcinoma, long-term outlook for most patients remains poor. Surgical resection is usually prohibited because of its local invasiveness, and most patients can only be managed by palliative drainage. Recently, many surgeons have adopted a more aggressive resection with varying degrees of success. Several prognostic factors in bile duct carcinoma have been proposed; however, no reports have specifically focused on resected hilar cholangiocarcinoma and its prognostic survival factors using multivariate analysis.**
***Methods:*** **The clinical records and pathologic slides of 49 cases with resected hilar cholangiocarcinoma were reviewed retrospectively. Twenty clinical and laboratory parameters were evaluated for their correlation with postoperative morbidity and mortality, whereas 31 variables were evaluated for their significance with postoperative survival. Variables showing statistical significance in the first univariate analysis were included in the following multivariate analysis using stepwise logistic regression test for factors affecting morbidity and mortality and Cox stepwise proportional hazard model for factors influencing survival.**
***Results:*** **There were 5 in-hospital deaths, and the cumulative 5-year survival rate in 44 patients who survived was 14.9%, with a median survival of 14.0 months. Multivariate analysis disclosed that coexistent hepatolithiasis and lower serum asparate aminotransferase levels (90 U/L) had a significant low incidence of postoperative morbidity, whereas a serum albumin of less than 3 g/dL was the only significant factor affecting mortality. Regarding survival, univariate analysis identified eight significant factors: 1) total bilirubin 10 mg/dL, 2) curative resection, 3) histologic type, 4) perineural invasion, 5) liver invasion, 6) depth of cancer invasion, 7) positive proximal resected margin, and 8) positive surgical margin. However, multivariate analysis disclosed total bilirubin $\geq$ 10 mg/dL, curative resection, and histologic type as the three most significant independent variables.**
***Conclusions:*** **Surgical resection provides the best survival for bilar cholangiocarcinoma. An adequate nutritional support to increase serum albumin over 3 g/dL is the most important factor to decrease postoperative mortality. Moreover, preoperative biliary drainage to decrease jaundice and a curative resection with adequate surgical margin are recommended if longer survival is anticipated. Patients with well differentiated adenocarcinoma seem to survive longer compared to those with moderately or poorly differentiated tumors.**

*Keywords:* Hilar cholangiocarcinoma, liver resection, bile duct resection

### PAPER DISCUSSION

Su *et al.* have studied factors influencing surgical outcomes in hilar cholangiocarcinoma using univariate and multivariate analysis. 49 tumors were resected over a period of about 13 years in two hospitals in Taiwan, an average of about 4 per year or 2 per year per hospital, although most of the procedures were done in one hospital. One-half of the procedures were palliative, defined as having microscopically or macroscopically involved resection margins. How many patients had macroscopically involved versus microscopically involved margins is not stated. 5 patients died in the postoperative period, 4 in the curative group. The overall 5 year survival was 15% if one excluded the 5 postoperative deaths. 5 year survival in the curative group was about 35%, and would have been about 30% had postoperative deaths not been excluded. No patient who had a palliative resection survived 5 years.

All 49 patients were entered in the prognostic analysis. 18 risk factors were examined for their relation to morbidity and mortality by a univariate analysis in which the significance level was set at $p < 0.05$ level. Morbidity was reduced in patients with hepaticolithiasis and increased in patients with high AST and these factors persisted in the multivariate analysis. Low albumin and low hemoglobin were significantly related to mortality in the univariate analysis, but only the former variable persisted in the multivariate analysis. Although mortality rates were about 4 times higher (15.4% vs 4.3%) in patients over 65, age was not significantly related to either morbidity or mortality in this analysis. Eight variables were related to long term survival in the univariate analysis and elevated bilirubin, curative resection and histologic type were found to be significant in the multivariate analysis.

*Comments:* This is an interesting paper but there are a number of methodological and theoretical problems that render a number of the conclusions questionable.

The statistical methods adopted by the authors are well suited to analysis of large numbers of patients, but not to the small sample size in this study. Large numbers tend to assure that the data is representative of the population, whereas random non-representative events occurring in only a few patients may result in erroneous conclusions when the sample studied is small. Is the finding that hepaticolithiasis protects against morbidity one of these types of findings? Is it merely chance that a few patients without stones developed complications that led to this finding or is hepaticolithiasis really protective? The question is unanswerable, but much more confidence in the conclusion would be had if the sample size were large instead of small. Another problem with studies with small numbers is that they lack power. One only needs to look at the fact age was not found to be a significant factor in morbidity despite the large difference in mortality rates in patients under and over age 65. In other words small numbers renders these methods insensitive to variables that are truly significant and subject to concluding that truly non-significant variables are significant.

A second problem is that testing a large number of variables against a probability standard that approaches the number of variables being tested will predictably result in finding of significance simply by chance. For instance using the standard $p < 0.05$ means that there is a 1 in 20 chance that the finding of significance is due to chance. If, as in this study, one examines 18 risk factors against morbidity and mortality and use as probability due to chance a standard of 1 in 20, there is a good chance that significance will be found in one variable due to chance. Statisticians usually deal with this problem by lowering the $p$ value at which significance is declared from 0.05 by division of 0.05 by the square root of the number of variables tested. In this case an appropriate $p$ level would have been about 0.01 and not 0.05. Of course this puts great pressure on the data when the numbers are small. Only very striking and obvious factors would be significant – for instance if hepaticolithiasis was a protective factor then almost all patients without morbidity would have to have this factor present for it to be significant at the $p < 0.01$ level in this sample size.

A third problem relates to the fact that virtually all statistical estimates have confidence limits. These are critical to the interpretation of the data. One type of confidence limit applies to odds ratios. For instance one might find that the odds of dying from an operative procedure at age over 65 were three times as great as dying from the same procedure at age under 65 in a particular data set. Yet, as impressive as this figure is, it is not interpretable without the confidence limits of this estimate. In one scenario the confidence limits would include 1.0 (eg. 3.0 [0.5–6] confidence limits given in square brackets). 1.0 is the null hypothesis. It indicates no difference in odds since the value 0.5 means that it is possible that there is lesser chance of dying in the older group. These results say that the estimate of difference based on age includes the possibility that there is no difference and the data is not significant. Data of the form (3.0 [2.0–4.0]) excludes 1.0 and is significant. This data says that the probability of dying of the procedure in patients over 65 is between 2 and 4 times greater than in patients under 65. In the study under discussion the confidence limits of AST and hepaticolithiasis as given in Table V appear to include 1 and are therefore not significant in the multivariate analysis, as is stated in the text.

On another level one might ask what is the purpose of the survival analysis? Virtually all studies including this one show that a positive resection margin, *i.e.*, a palliative resection, is incompatible with long term survival. One does not need sophisticated statistical techniques to tell us this. In fact at this point are we not really interested in detecting prognostic factors that affect long term survival in patients who have had curative resections only? In that case one should only enter patients who have had curative resection. Presumably the multivariate nature of the model would preclude false outcomes based on the fact that palliative and curative respections were analyzed together. Indeed curative resection was one of 3 factors affecting long term outcome in the multivariate analysis. Another factor, bilirubin >10 mg% was associated with poorer outcome and presumably this was not due to a tendency for patients with higher bilirubin to have palliative resection, since bilirubin was significant in the multivariate analysis. Yet again one must look at the confidence limits [0.17–0.99] which means the data was barely significant. Greater confidence would be gained by simply evaluating the effect of bilirubin in curatively resected cases.

Here's another point regarding multivariate analyses that is not widely appreciated. Histologic grade was related to long term outcome. Why wasn't perineural invasion or depth of invasion of tumor found to be significant as they have been in other studies that the authors cite. In order to understand this one must understand how the multivariate analysis works. Each independent variable (eg. histologic grade, perineural invasion) is examined in turn to see how well it explains the variability of the results for the independent variable (long term survival). When the factor that best explains the variability is identified it is declared to be related to the factor of interest, provided that the relationship is significant. Other factors are then tested for their ability to explain the residual variability in the data, *i.e.*, the data not explained by the factor that best explains the data. It is this part of the test that eliminates independent variables that are related to other independent variable and not to the dependent variable. For instance in a study on the cause of lung cancer one would find that while smoking and alcohol might be related to the development of lung cancer in a univariate analysis only smoking is so in a multivariate analysis. The finding in the univariate analysis is due to the fact that smokers also tend to be drinkers. There is however a fallout for this necessary procedure, which is that closely related independent variables, which are truly related to the dependent variable, tend to predict the same type of variability and the one which does it best will depend to an extent on chance, especially in small studies. The result is that if histologic grade is found to explain variability better than perineural invasion even by a tiny margin it will be the selected factor, but because they explain the same variability, perineural invasion will not explain residual variability well, and will be found not to be significant. If the study is repeated it is not unlikely that perineural invasion could eke out a numerical victory over histologic grade and the opposite occur – it would be the significant factor and not histologic grade. In other words if one tests many closely related factors (such as factors related to tumor aggressiveness) it would not be surprising if they were all positive in the univariate analysis, only one was positive in the multivariate analysis and that the one which was positive would vary from study to study because there is no absolute measure of tumor aggressiveness and what comes out at the top in the analysis is largely random. This is just what has happened in this field and also in other prognostic studies such as studies examining prognosis of hepatic colorectal cancer metastases.

What is to be done? Large co-operative studies are needed since very few institutions can mount a study with the numbers required. Beyond that we probably need more help in understanding the limits of resolution of statistical methods.

Steven M. Strasberg, MD
Professor of Surgery
Washington School of Medicine
1 Barnes Hospital Plaza
Box 8109
St Louis, M0 63110
United States of America

